# Hotspots and trends in peripheral facial paralysis treatment: A bibliometric analysis

**DOI:** 10.1097/MD.0000000000050785

**Published:** 2026-09-18

**Authors:** Fan Dai, Fangyuan Xu, Xiaoyu Han, Peijia Hu, Hongliang Cheng

**Affiliations:** a The First Clinical Medical School, Anhui University of Chinese Medicine, Hefei, Anhui, China; b Department of Neurology, The Second Affiliated Hospital of Anhui University of Chinese Medicine, Hefei, Anhui, China.

**Keywords:** bibliometric analysis, CiteSpace, peripheral facial paralysis, therapy, VOSviewer

## Abstract

**Background:**

Peripheral facial paralysis (PFP) is a nonspecific inflammatory disease of the facial nerve, and the resulting sequelae seriously affect social function and mental health. Bibliometric analysis determines the focus and status of current research. However, no comprehensive bibliometric study has yet focused on PFP treatment.

**Methods:**

Relevant publications from 2004 to 2024 were retrieved from the Web of Science. Visualization and bibliometric analyses were conducted using VOSviewer, CiteSpace, and Scimago Graphica to explore collaborative networks among countries, institutions, and authors, as well as key journals and references.

**Results:**

A total of 2426 publications were identified, authored by 10,111 researchers from 2749 institutions across 84 countries/regions. Publication output has shown a general upward trend. The United States led in publication volume and international collaborations. The institution and author with the most publications were Kyung Hee University and Hadlock TA, respectively. *Plastic and Reconstructive Surgery* ranked highest in both publication count and citations, while Terzis JK was the most frequently co-cited author. Over the years, the research hotspots in PFP treatment shifted from antivirals, steroids, and muscle transplantation therapies to include nerve transplantation, acupuncture, electrical stimulation, and other rehabilitation therapies.

**Conclusion:**

This analysis captured the research hotspots and frontiers in PFP therapy. This study highlights the developmental trajectory and emerging trends in PFP therapy, providing a comprehensive reference for future research directions in the field.

## 1. Introduction

Peripheral facial paralysis (PFP) is a nonspecific inflammatory reaction involving the facial nerve canal, characterized by paralysis of the facial muscle.^[1]^ The condition mostly manifests as a crooked mouth and the inability to raise eyebrows, close eyes, and show teeth. Common causes leading to PFP include Bell palsy, Ramsay Hunt syndrome, and trauma, among others.^[2]^ Research indicates that the annual incidence rate of PFP is approximately 11 to 40 per 100,000 individuals, with similar rates among men and women; however, slightly higher rates have been reported in middle-aged and older populations.^[3,4]^ Despite the relatively good prognosis of PFP, about 25% of patients do not recover completely. In such cases, the lasting facial asymmetry significantly impacts their social activities and quality of life, leading to anxiety and depression.^[5,6]^ In summary, facial muscle paralysis and its recurrence influence patients’ mental health and quality of life, imposing a significant burden on families and society.

Nonetheless, the pathogenesis of PFP remains incompletely understood.^[7]^ With the advancement and development of technology, PFP patients have more treatment options, including medications such as corticosteroids and antiviral drugs, surgical interventions, physical therapy, acupuncture, etc.^[8,9]^ However, the efficacy of the aforementioned treatment methods remains controversial.^[10]^ Notably, a bibliometric analysis on the current status, hotspots, and future trends in PFP treatment is lacking. Therefore, a bibliometric study on the field of PFP therapy will assist in exploring the development context, research focus, and future prospects of this area, thereby enhancing the accuracy of clinical treatment.

Bibliometric analysis is an approach that quantitatively extracts, counts, and analyzes data from articles.^[11]^ Data such as titles, authors, abstracts, keywords, and publication years are collected to provide a visual analysis, revealing the development trends and research hotspots in the subject area.^[12,13]^ Consequently, this research employs VOSviewer and CiteSpace software for the 1st time to conduct a bibliometric analysis of publications concerning PFP therapy over the last 2 decades.

## 2. Materials and methods

### 2.1. Literature source and search strategy

**Figure 1.**
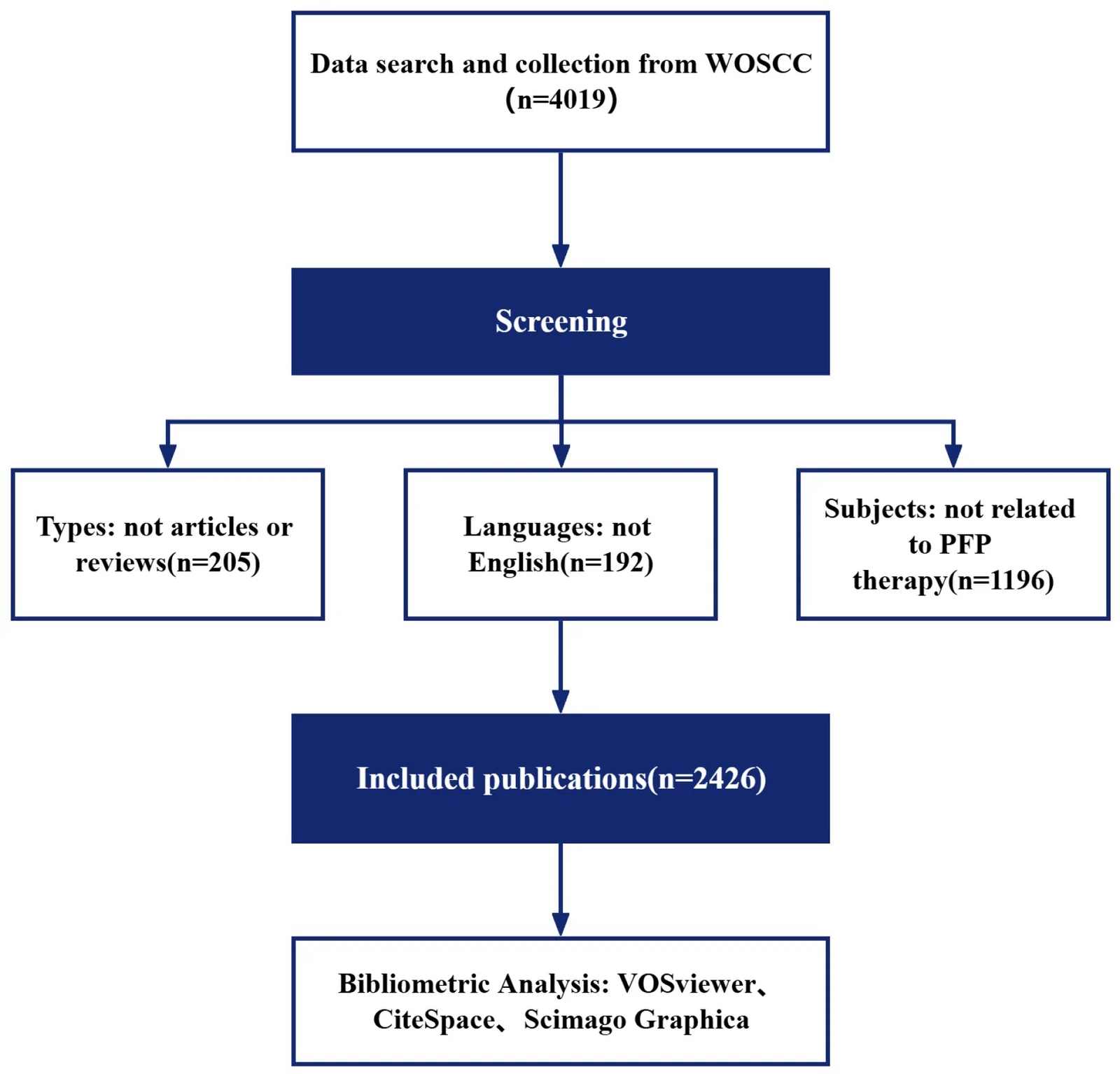
Flowchart of the literature inclusion process. PFP = peripheral facial paralysis, WOSCC = Web of Science Core Collection.

This research analyzed studies from the Web of Science Core Collection (WOSCC) database, gathering all articles pertaining to PFP therapy published before November 3, 2024. The literature search was performed using the following terms: TS = (“facial paralysis” OR “facial palsy” OR “peripheral facial paralysis” OR “Bell palsy” OR “Bell facial paralysis” OR “idiopathic facial paralysis” OR “lower motor neuron facial palsy” OR “idiopathic acute facial neuropathy”) AND TS = (“treatment*” OR “cure*” OR “treat*” OR “therapy*” OR “therapeutic*” OR “management” OR “drug”). The publication types were restricted to article and review, with the language set to English, covering the period from 2004 to 2024. After duplicate records were removed, titles and abstracts were screened for relevance, with full texts reviewed when eligibility could not be determined from the bibliographic record. Records were excluded if they did not primarily concern PFP, did not address its treatment or clinical management, or lacked the bibliographic information required for the planned analyses. When full-text assessment was necessary, the article was sought through institutional subscriptions, open-access sources, and interlibrary loan before being considered unavailable. Bibliographic information was regarded as insufficient only when missing data, such as authorship, publication year, source title, keywords, or cited references, prevented reliable identification or analysis of the record. A missing abstract alone was not a reason for exclusion if eligibility could be established from the remaining information. Eligible records were exported in plain-text format using the “Full Record and Cited References” option and imported into CiteSpace and VOSviewer. The final dataset comprised 2426 publications, including 2100 articles and 326 reviews. The flowchart of literature screening is illustrated in Figure 1.

### 2.2. Data analysis

Data visualization analysis was conducted using VOSviewer V1.6.20 (Centre for Science and Technology Studies, Leiden University, Leiden, the Netherlands), CiteSpace V6.3 (Drexel University, Philadelphia), and Scimago Graphica V1.0.46 (SCImago Lab, Spain) software. Subsequently, Microsoft Excel 2021 was utilized to summarize the annual number of publications and generate area charts for data analysis. The contribution and connections of publication volumes between countries were analyzed using Scimago Graphica V1.0.46.^[14]^ VOSviewer and CiteSpace are both mapping software tools used for bibliometric analysis.^[15,16]^ VOSviewer V1.6.20 was employed for the network co-occurrence analysis of institutions, authors, and journals. The clustering, citation bursts, and timeline view of keywords and references were analyzed by CiteSpace V6.3. Visual network maps are composed of nodes and lines, with the size of the node representing frequency and the thickness of the line indicating the strength of the link. These software tools were used to analyze the research and development trends pertaining to PFP therapy from diverse perspectives.

### 2.3. Ethical review

Ethics committee approval and informed consent were not required because this bibliometric study analyzed only published bibliographic records retrieved from the WOSCC and involved no human participants, identifiable personal data, or animal experiments.

## 3. Results

### 3.1. Trends in publication output

**Figure 2.**
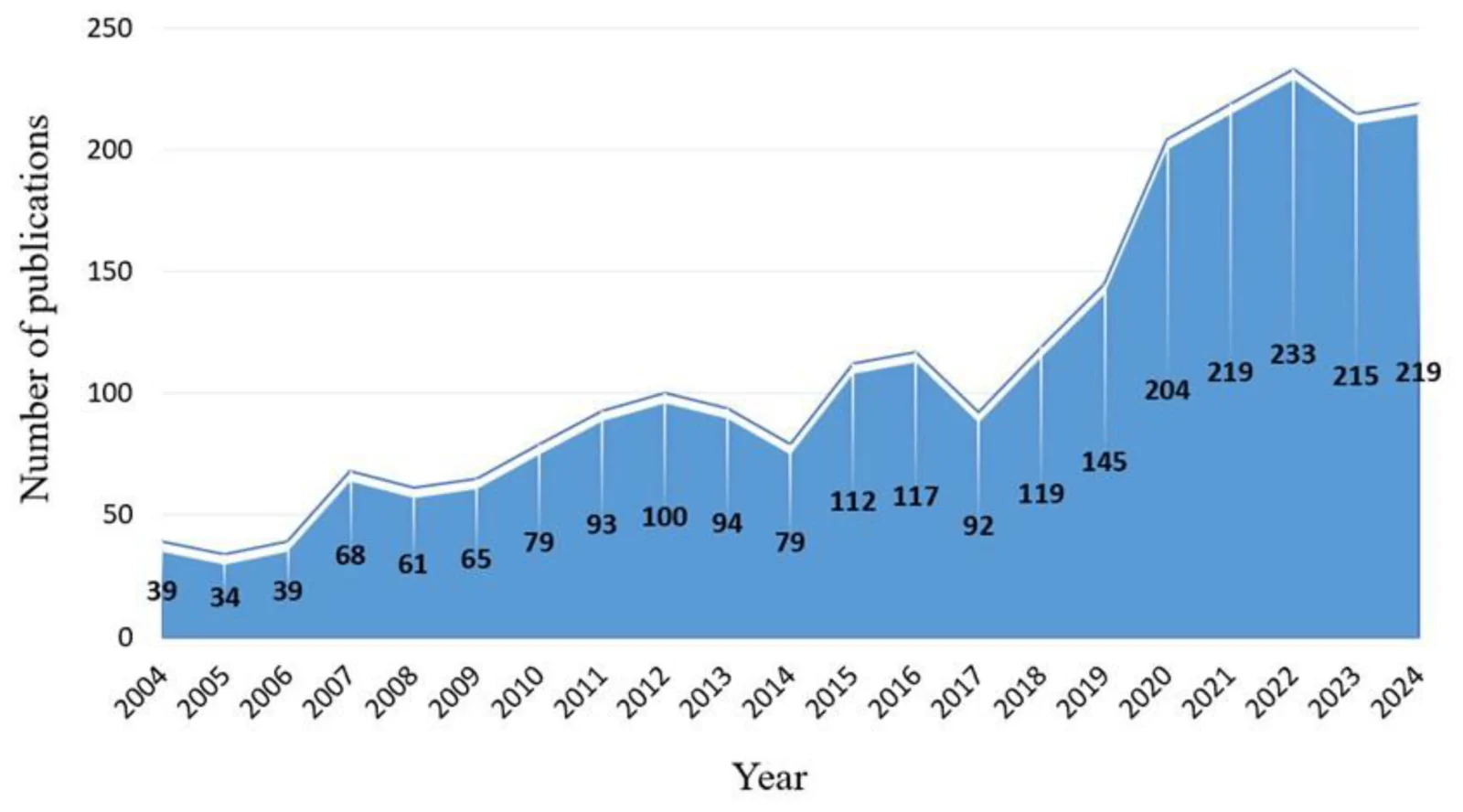
Annual publication volume and trend graph.

As of the search date, this study included 2426 publications that met the criteria, consisting of 2100 articles and 326 reviews. Figure 2 illustrates the annual publication count pertaining to PFP therapy over the last 2 decades. Despite fluctuations in annual output, an overall upward trend was observed, particularly from 2017 to 2022. In 2004, only 39 articles were published concerning PFP therapy. However, as awareness of PFP increased, additional studies were conducted on this topic, reaching a peak of 233 publications in 2022. This surge may also be related to clinical case reports following COVID-19 vaccination.^[17]^ The above trend indicates growing attention to the field of PFP therapy.

### 3.2. Countries and institutions

**Figure 3.**
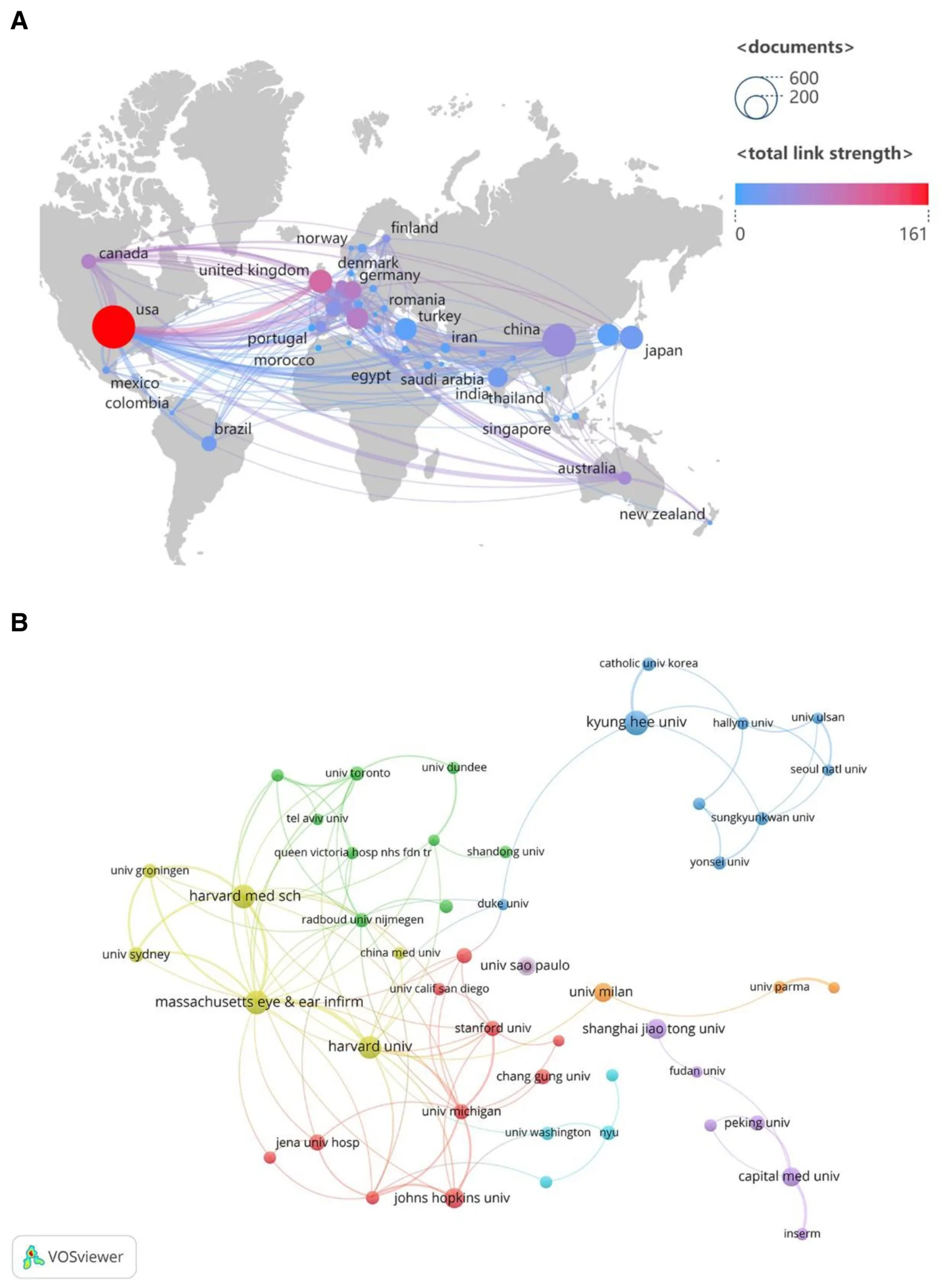
Analysis of countries and institutions. (A) World map of publications and international collaboration graph. (B) Institutional collaboration network graph for PFP treatment. PFP = peripheral facial paralysis.

Over the last 20 years, 84 countries have contributed to research in the PFP therapy area. The world map of publications was drawn using Scimago Graphica (Fig. 3A). In Figure 3A, each circle represents a country; the size of the circle is positively correlated with the number of publications, and the color of the lines indicates the strength of cooperation between countries, ranging from blue to red, representing low to high cooperation intensity. Table 1 lists the top 10 countries in terms of publication count and total link strength. As displayed in Table 1, the United States was the most productive country with 570 publications (23.50%), followed by China (349 publications, 14.39%), Japan (171 publications, 7.05%), the United Kingdom (165 publications, 6.80%), and South Korea (148 publications, 6.10%). The United States has engaged in more active cooperation with other countries in this field, with a total link strength (TLS) of 175, followed by the United Kingdom (TLS: 107), Germany (TLS: 77), Italy (TLS: 76), and Canada (TLS: 73). Although China currently has a higher volume of publications, its cooperation with other countries is lacking. In the future, deeper international exchanges and cooperation are expected in the field of PFP treatment.

**Table 1:** Top 10 countries in terms of publication count and total link strength.

Rank	Country	Documents	Rank	Country	TLS
1	USA	570	1	USA	175
2	China	349	2	United Kingdom	107
3	Japan	171	3	Germany	77
4	United Kingdom	165	4	Italy	76
5	South Korea	148	5	Canada	73
6	Italy	144	6	Netherlands	65
7	Turkey	134	7	Australia	59
8	India	123	8	Switzerland	55
9	Germany	102	9	China	52
10	France	82	10	Egypt	43

TLS = total link strength.

This study analyzed 2426 documents from 2749 institutions and used VOSviewer to plot the institutions that published >10 articles (Fig. 3B). As shown in Figure 3B, the diameter of each circle corresponds to the number of publications, while the thickness of the connecting lines reflects the intensity of collaborative relationships. Table 2 lists the top 10 institutions globally in terms of output volume. The top 10 institutions account for 13.06% of the total global output, with the United States having the highest number of institutions. Kyung Hee University contributed the most with 43 papers, followed by Harvard Medical School (41 papers) and Massachusetts Eye and Ear Institute (40 papers). Massachusetts Eye and Ear Institute showed the highest number of collaborations, followed closely by Harvard University (TLS: 31) and Harvard Medical School (TLS: 30). However, there is still a lack of collaboration among these top institutions, especially with Universidade de São Paulo.

**Table 2:** Top 10 institutions in terms of output volume, affiliated countries, and total link strength.

Rank	Institution	Country	Documents	TLS
1	Kyung Hee University	South Korea	43	10
2	Harvard Medical School	USA	41	30
3	Massachusetts Eye and Ear Institute	USA	40	46
4	Harvard University	USA	39	31
5	Johns Hopkins University	USA	28	13
6	Shanghai Jiao Tong University	China	28	1
7	Capital Medical University	China	27	12
8	University of Milan	Italy	26	2
9	Universidade de São Paulo	Brazil	26	0
10	Jena University Hospital	Germany	19	6

TLS = total link strength.

### 3.3. Authors and co-cited authors

**Figure 4.**
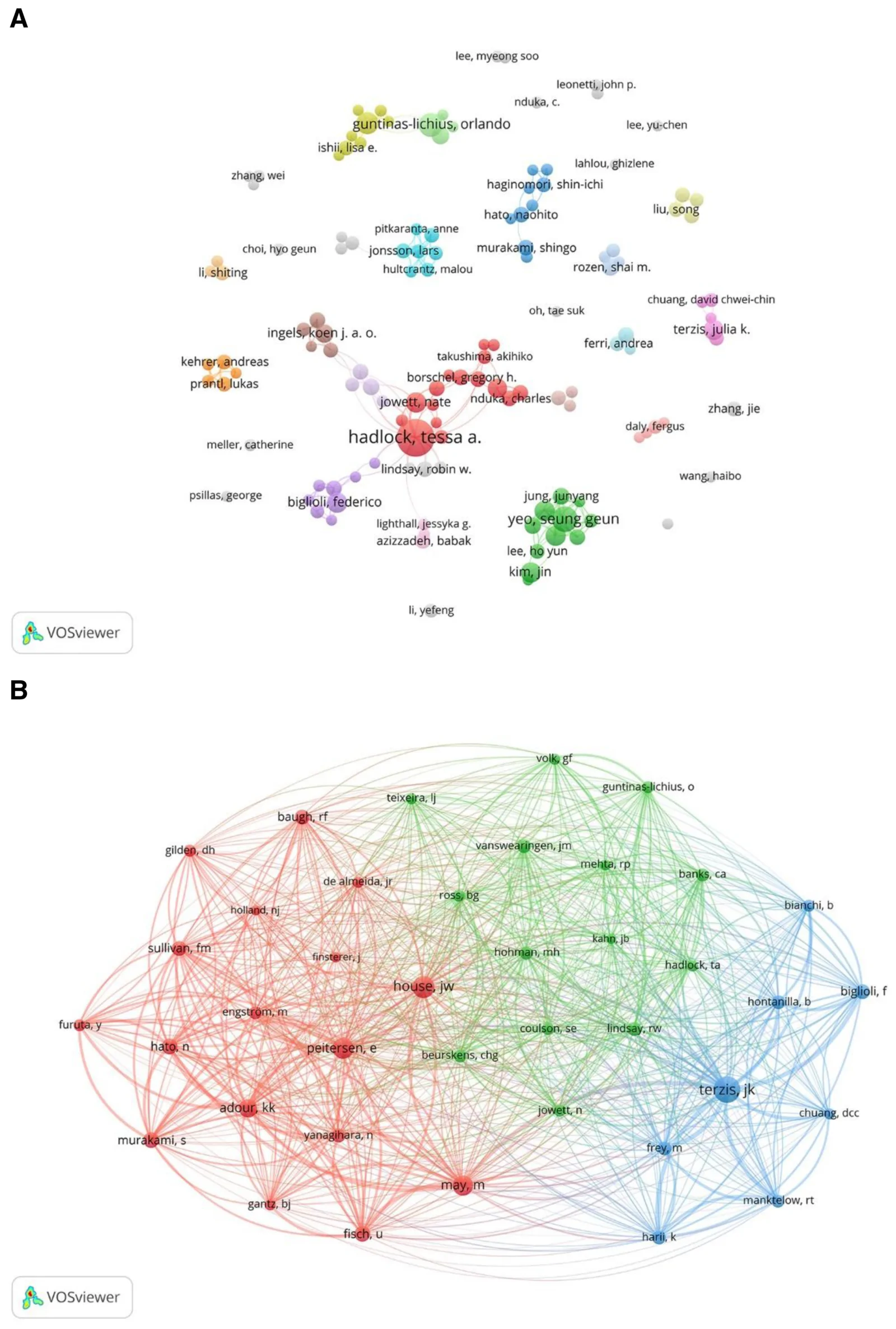
Analysis of authors and co-cited authors. (A) Author co-occurrence network. (B) Co-cited author co-occurrence network.

From the 2426 documents reviewed, a total of 10,111 primary authors and 26,220 collaborators were engaged in investigations concerning PFP therapy. Figure 4A illustrates the co-authorship network comprising authors who have published >5 articles, while Table 3 lists the top 10 authors with the highest output and citations. In terms of the number of publications, the most prolific author was Hadlock TA, with 53 publications, followed by Yeo SG (26 publications), Guntinas-Lichius O (22 publications), Byrne PJ (19 publications), and Biglioli F (16 publications). Hadlock TA stands out with the highest number of publications and the most citations at 1848, with Terzis JK ranking 2nd with 585 citations, followed by Guntinas-Lichius O (566 citations), Lindsay RW (448 citations), and Byrne PJ (442 citations). As shown in Figure 4A, a few solid core author clusters have appeared, but the opportunities for collaboration among these clusters still need to be investigated.

**Table 3:** Top 10 authors with the highest number of publications and citations.

Rank	Author	Documents	Rank	Author	Citations
1	Hadlock TA	53	1	Hadlock TA	1848
2	Yeo SG	26	2	Terzis JK	585
3	Guntinas-Lichius O	22	3	Guntinas-Lichius O	566
4	Byrne PJ	19	4	Lindsay RW	448
5	Biglioli F	16	5	Byrne PJ	442
6	Kim J	16	6	Yeo SG	409
7	Terzis JK	16	7	Jowett N	360
8	Byun JY	15	8	Murakami S	55
9	Jowett N	15	9	Hadlock T	52
10	Kim SH	15	10	Biglioli F	43

Figure 4B shows the co-occurrence network of authors with >100 co-citations. Table 4 lists the top 10 co-cited authors with the highest number of citations and TLS. The most frequently co-cited author was Terzis JK (696 times), whose most cited article described a microvascular submental nerve transfer to improve facial function in patients with facial paralysis.^[18]^ House JW ranked 2nd with 484 citations, followed by Peitersen E with 433 citations, May M with 410 citations, and Adour KK with 348 citations. Terzis JK not only had the highest number of co-citations but also showed the closest collaboration with other authors (TLS: 4395), followed by May M (TLS: 3123), Peitersen E (TLS: 2986), Adour KK (TLS: 2967), and House JW (TLS: 2795).

**Table 4:** Top 10 co-cited authors with the highest number of citations and total link strength.

Rank	Co-cited author	Citations	Rank	Co-cited author	TLS
1	Terzis JK	696	1	Terzis JK	4395
2	House JW	484	2	May M	3123
3	Peitersen E	433	3	Peitersen E	2986
4	May M	410	4	Adour KK	2967
5	Adour KK	348	5	House JW	2795
6	Hato N	235	6	Hato N	1865
7	Murakami S	235	7	Fisch U	1816
8	Fisch U	233	8	Lindsay RW	1807
9	Sullivan FM	221	9	Sullivan FM	1748
10	Biglioli F	216	10	Murakami S	1737

TLS = total link strength.

### 3.4. Journals and co-cited journals

**Figure 5.**
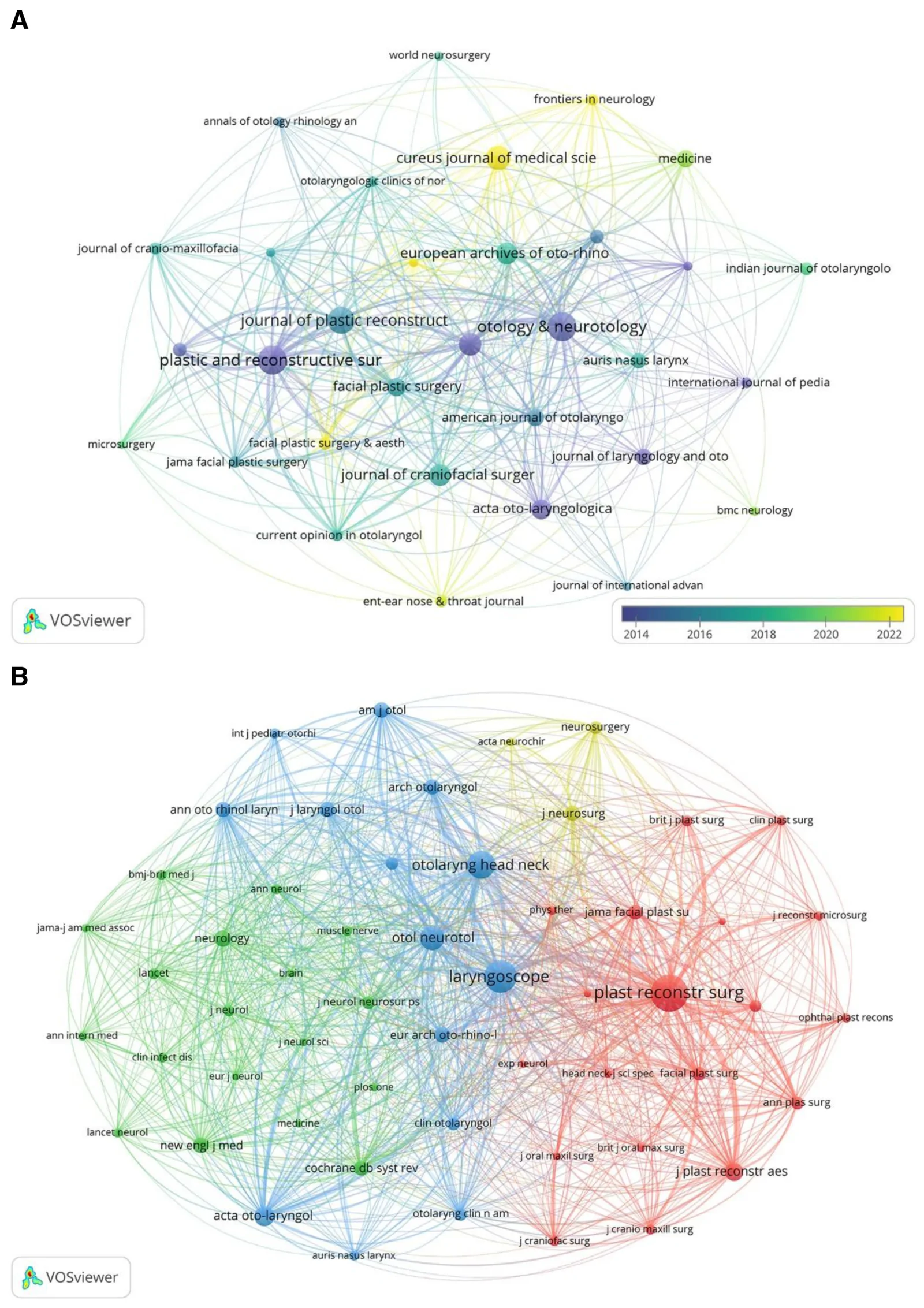
Analysis of journals and co-cited journals. (A) The co-occurrence network map of journals. (B) The network map of co-cited journals.

A total of 2426 documents published in 712 journals were included in this study. Table 5 lists the top 10 journals with the most publications. The journal with the highest number of publications on PFP therapy is *Plastic and Reconstructive Surgery* (83 articles), followed by *Otology & Neurotology* (81 articles) and *Journal of Plastic Reconstructive and Aesthetic Surgery* (75 articles). Among the top 10 journals, only 2 belonged to the *Q*1 journal citation reports category, with *Plastic and Reconstructive Surgery* having the highest impact factor (IF). The co-occurrence network of journals regarding PFP therapy is depicted in Figure 5A. A visualization map of 32 journals containing >13 articles each was generated, reflecting the changes in journal inclusion over time. Purple nodes represent journals with earlier research times, while yellow nodes indicate later research times. Recently, a growing number of journals have published articles in this field, such as *Cureus Journal of Medical Science*, *Facial Plastic Surgery & Aesthetic Medicine*, and *Frontiers in Neurology.*

**Table 5:** Top 10 journals with the most publications.

Rank	Journal	Documents	2024 IF	JCR
1	*Plastic and Reconstructive Surgery*	83	3.2	*Q*1
2	*Otology & Neurotology*	81	1.9	*Q*2
3	*Journal of Plastic Reconstructive and Aesthetic Surgery*	75	2.0	*Q*2
4	*Cureus Journal of Medical Science*	64	1.0	*Q*3
5	*Laryngoscope*	55	2.2	*Q*1
6	*Journal of Craniofacial Surgery*	54	1.0	*Q*3
7	*European Archives of Oto-Rhino-Laryngology*	52	1.9	*Q*2
8	*Acta Oto-Laryngologica*	45	1.2	*Q*3
9	*Facial Plastic Surgery*	40	1.1	*Q*3
10	*American Journal of Otolaryngology*	38	1.8	*Q*2

IF = impact factor, JCR = journal citation reports.

A total of 8307 co-cited journals have contributed to the research of PFP therapy. Figure 5B shows the co-citation network of 49 co-cited journals with >200 citations, which are divided into 5 distinct clusters of different colors. The largest red cluster is focused on the fields of medicine, internal medicine, and clinical neurology, while the most frequently cited journals are concentrated in the green and blue clusters. Table 6 lists the 10 most referenced journals, with over 50% cited >1000 times, and 5 of the top 10 journals belonging to the *Q*1 journal citation reports division. The most frequently cited and influential journal was *Plastic and Reconstructive Surgery* (4015 times, IF = 3.2), followed by *Laryngoscope* (3164 times, IF = 2.2) and *Otolaryngology-Head and Neck Surgery* (2342 times, IF = 2.7).

**Table 6:** Top 10 co-cited journals with the most citations.

Rank	Co-cited journal	Citations	2024 IF	JCR
1	*Plastic and Reconstructive Surgery*	4015	3.2	*Q*1
2	*Laryngoscope*	3164	2.2	*Q*1
3	*Otolaryngology-Head and Neck Surgery*	2342	2.7	*Q*1
4	*Otology & Neurotology*	1724	1.9	*Q*2
5	*Acta Oto-Laryngologica*	1201	1.2	*Q*3
6	*Journal of Plastic Reconstructive and Aesthetic Surgery*	1008	2.0	*Q*2
7	*Neurology*	755	8.4	*Q*1
8	*New England Journal of Medicine*	748	96.3	*Q*1
9	*Journal of Laryngology and Otology*	721	1.1	*Q*3
10	*Journal of Neurosurgery*	721	3.5	*Q*1

IF = impact factor, JCR = journal citation reports.

### 3.5. Keywords

**Figure 6.**
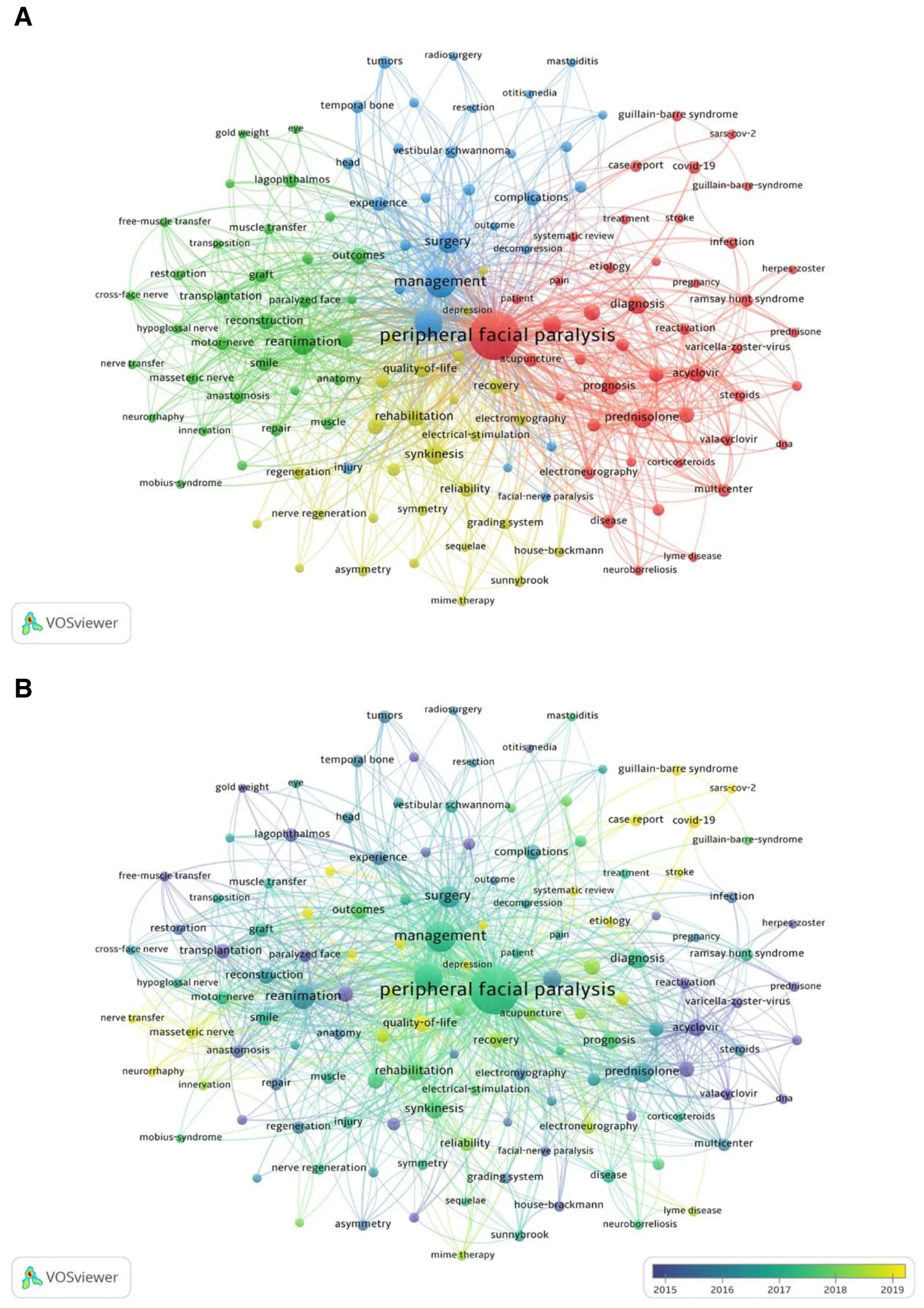
Analysis of keywords. (A) The co-occurrence and clustering map of keywords concerning PFP therapy. (B) The overlap visualization map of keywords related to PFP therapy. PFP = peripheral facial paralysis.

Keywords summarize the theme and main content of an article. Hence, keyword analysis can reflect the frontier directions in this field.^[19]^ This study used VOSviewer to generate a co-occurrence and cluster diagram of keywords, as shown in Figure 6A. The keywords in the figure are divided into 4 prominent clusters. The red cluster focuses on the etiology, diagnosis, and pharmacological treatment of PFP, while the blue cluster primarily addresses the surgical treatment of PFP. The green cluster mainly involves nerve and muscle transplantation techniques that promote facial function recovery, and the yellow cluster pertains to scales for evaluating the effectiveness of PFP treatments and rehabilitation methods. As shown in Table 7, the most common keywords were “peripheral facial paralysis” (1581 times), “management” (470 times), “facial nerve” (351 times), “reanimation” (250 times), and “surgery” (179 times). Peripheral facial paralysis also exhibited the highest TLS of 5013. As shown in Figure 6B, the visualization of keywords demonstrated the variations in research trends in the time dimension. The purple and blue nodes indicate an earlier emergence of keywords, showing that scholars have extensively researched antiviral therapy, muscle transplantation, and the efficacy evaluation in PFP therapy. The green and yellow keywords emerged later, showing that the subsequent therapeutic research focused on corticosteroids, nerve transplantation, acupuncture, electrical stimulation therapy, and various rehabilitation methods.

**Table 7:** Top 10 keywords in terms of frequency related to PFP therapy.

Rank	Keyword	Frequency	TLS
1	peripheral facial paralysis	1581	5013
2	management	470	1768
3	facial nerve	351	1456
4	reanimation	250	1339
5	surgery	179	748
6	prednisolone	145	752
7	synkinesis	140	687
8	diagnosis	134	432
9	children	123	473
10	rehabilitation	111	561

TLS = total link strength.

### 3.6. References

**Figure 7.**
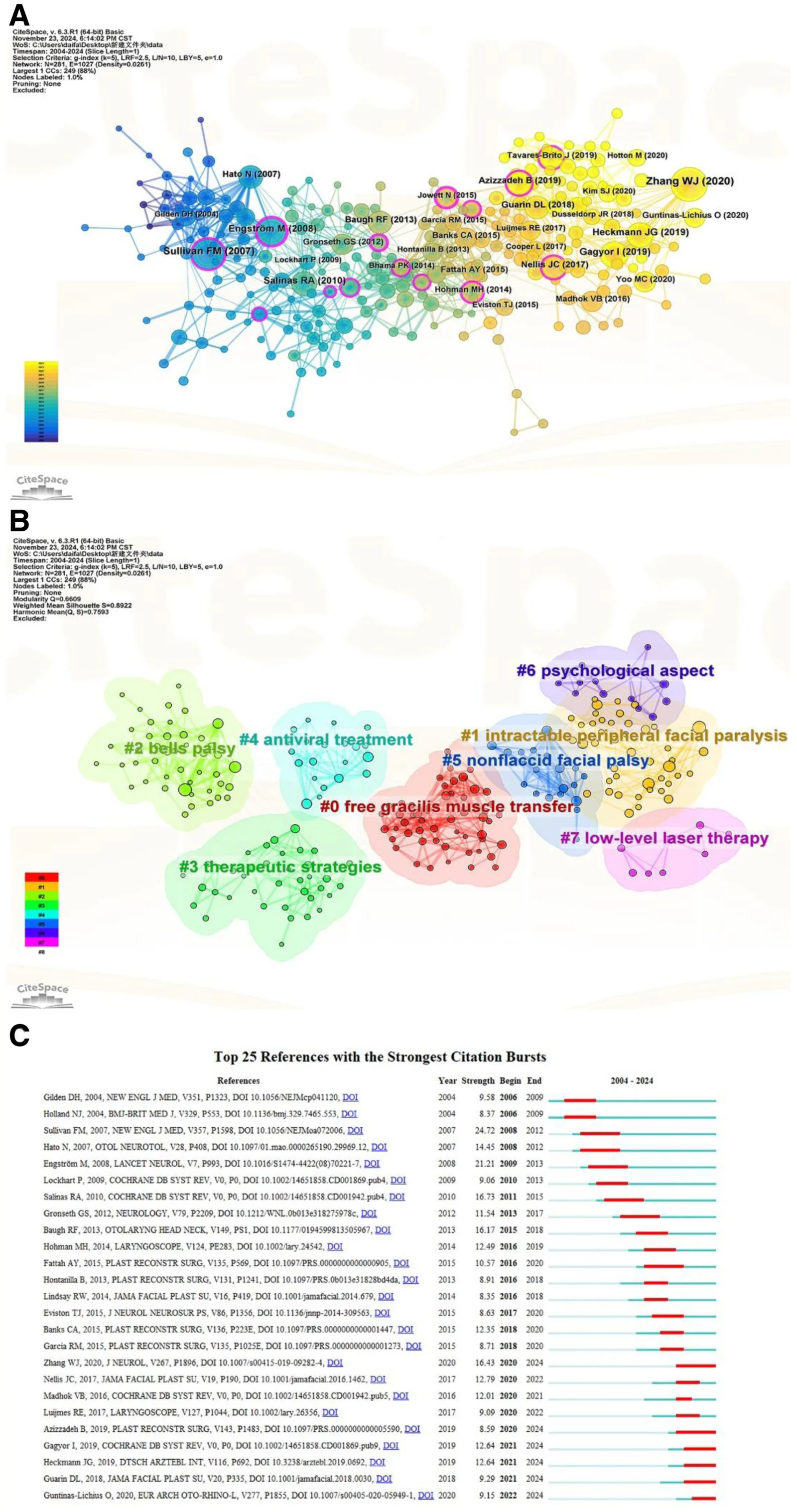
Analysis of references. (A) The visualized graph of co-cited references related to PFP treatment. (B) The co-citation analysis of reference clusters for PFP therapy. (C) The top 25 cited references with the strongest citation burst in the field of PFP therapy. PFP = peripheral facial paralysis.

Co-citation reference analysis evaluates co-occurrence through the number of commonly cited articles, thereby exploring the development of this field.^[20]^ To analyze the main references and their timeline in the field of PFP treatment, this study employed CiteSpace to create visual maps from different perspectives. Figure 7A presents a co-citation map of the common reference literature, with nodes displayed as citation rings. The size of the rings is proportional to the frequency of keyword occurrences, and the color of the rings represents the active time period for the keywords. In addition, Table 8 lists the 10 most commonly cited references. Each of these references has been cited over 90 times, including 2 reviews, 7 clinical studies, and 1 guideline. The most cited article is “Facial Nerve Grading System,” published in the journal *Otolaryngology-Head and Neck Surgery*. This article introduces the House–Brackmann (H-B) grading scale, developed by House JW and Brackmann DE for facial function scoring in PFP cases, which remains extensively utilized in clinical practice.^[21]^

**Table 8:** The top 10 co-cited references in the field of PFP treatment.

Rank	Citations	Title	Authors	Journal	Year
1	428	Facial nerve grading system	House JW, Brackmann DE	*Otolaryngology-Head and Neck Surgery*	1985
2	230	Bell palsy: the spontaneous course of 2500 peripheral facial nerve palsies of different etiologies	Peitersen E, et al.	*Acta oto-Laryngologica*	2002
3	206	Early treatment with prednisolone or acyclovir in Bell palsy	Sullivan FM, et al.	*New England Journal of Medicine*	2007
4	178	Clinical practice guideline: Bell palsy	Baugh RF, et al.	*Otolaryngology-Head and Neck Surgery*	2013
5	152	Prednisolone and valaciclovir in Bell palsy: a randomized, double-blind, placebo-controlled, multicentre trial	Eengström M, et al.	*Lancet Neurology*	2008
6	151	Bell palsy and herpes simplex virus: identification of viral DNA in endoneurial fluid and muscle	Murakami S, et al.	*Annals of Internal Medicine*	1996
7	147	Development of a sensitive clinical facial grading system	Ross BG, et al.	*Otolaryngology-Head and Neck Surgery*	1996
8	120	Bell palsy	Gilden DH, et al.	*New England Journal of Medicine*	2004
9	110	Validation of a patient-graded instrument for facial nerve paralysis: the FaCE scale	Kahn JB, et al.	*Laryngoscope*	2001
10	103	Etiology, diagnosis, and management of facial palsy: 2000 patients at a facial nerve center	Hohman MH, et al.	*Laryngoscope*	2014

PFP = Peripheral facial paralysis.

As shown in Figure 7B, the 34,746 references in this research field are divided into 8 clusters, with *Q* = 0.6609 and *S* = 0.8922, indicating the stability and persuasive power of the clustering structure. The largest cluster concerned free gracilis muscle transfer. Clusters #3 therapeutic strategy, #4 antiviral treatment, and #7 low-level laser therapy primarily focused on the related treatment methods of PFP. Clusters #1 intractable peripheral facial paralysis, #2 Bell palsy, and #5 nonflaccid facial palsy involved different types of PFP, while #6 psychological aspect explored the prognosis of PFP.

Figure 7C presents the 25 most prominent reference sources exhibiting the strongest citation burst intensities. The article “Early treatment with prednisolone or acyclovir in Bell palsy” published by Sulivan FM in 2007 demonstrated the strongest citation. This article confirmed the efficacy of prednisolone in the early treatment of Bell palsy but found no benefits in using acyclovir in the early stage of Bell palsy.^[22]^ The paper “Prednisolone and valaciclovir in Bell palsy: a randomized, double-blind, placebo-controlled, multicentre trial” by Engström M and others, published in 2008, showed the 2nd-highest impact in the cited burst analysis. This large-sample, multicenter, randomized, double-blind, placebo-controlled trial revealed that prednisolone could shorten the time for complete recovery in patients with Bell palsy, whereas valaciclovir had little effect on facial recovery.^[23]^ The article published by Salinas RA et al reported that the use of corticosteroids in the treatment of Bell palsy has a significant effect, ranking 3rd in the intensity of citation burst.^[24]^

## 4. Discussion

### 4.1. Basic information

Approximately 30% of patients with PFP experience persistent facial nerve dysfunction, which can substantially impair social functioning and quality of life,^[25]^ while recurrence occurs in approximately 5% to 7% of patients.^[1]^ These considerable long-term burdens have stimulated growing scientific interest in PFP. Bibliometric analysis can characterize the temporal evolution of a research field by constructing network-based knowledge maps and identifying developmental trends and emerging topics. To our knowledge, this is the 1st bibliometric analysis specifically focused on PFP therapy. It included 2426 publications produced by 10,111 researchers from 2749 institutions across 84 countries or regions between 2004 and 2024. Annual publication output showed an overall but fluctuating upward trajectory over the past 2 decades and peaked in 2022 (Fig. 2), indicating sustained expansion of this research field. This growth likely reflects increasing recognition of the long-term sequelae, impaired quality of life, and psychosocial burden associated with PFP, which has generated a continuing need for long-term management strategies. Concurrent advances in pharmacotherapy, microsurgical dynamic reanimation, nerve and muscle transfer, botulinum toxin injection, and rehabilitation have further broadened the scope of PFP treatment research.

Among the contributing countries, the United States was the largest contributor, ranking 1st in both publication output and TLS. Major US institutions, including Harvard Medical School and Massachusetts Eye and Ear, also made substantial contributions, and 4 of the 10 most productive institutions were located in the United States. This leadership may be attributable to its well-established otologic, plastic and reconstructive surgical, and multidisciplinary facial nerve centers, together with sustained research funding and infrastructure supporting longitudinal cohorts and multicenter collaboration. China and Japan also ranked among the leading contributors, possibly reflecting increasing research investment, large clinical populations, and active investigation of rehabilitation and nonpharmacological interventions. Notably, China ranked 2nd in publication output but only 9th in TLS (Table 1 and Fig. 3A). This discrepancy should not be interpreted as indicating lower research quality, because publication output and network connectivity represent distinct dimensions of national scientific contribution. Rather, it suggests that China’s rapidly increasing research productivity has not yet been matched by comparable integration into the international collaboration network. This pattern may be related to regionally concentrated research networks, language and database-indexing effects, and differences in clinical practice. Future multinational studies and greater resource sharing may improve the international representativeness, comparability, and transferability of evidence concerning PFP treatment.

At the institutional level, Kyung Hee University was the most productive institution, with 43 publications, whereas Massachusetts Eye and Ear had the highest institutional TLS (Table 2 and Fig. 3B). These findings suggest 2 broad forms of institutional leadership: output-oriented specialist centers that generate sustained research within particular therapeutic traditions and network-oriented hub institutions that connect multiple clinical specialties and research groups. Notably, the 10 most productive institutions collectively contributed only 13.06% of all publications, indicating that research output was widely distributed rather than dominated by a small number of centers. Such decentralization may promote diversity in therapeutic approaches and methodological perspectives. However, the relatively weak connections among some highly productive institutions may also contribute to inconsistencies in patient classification, treatment protocols, and outcome measurement. Strengthening multicenter and international collaboration is therefore particularly important for generating comparable evidence across pharmacological, surgical, and rehabilitative interventions.

Author analysis identified Hadlock TA as both the most productive and most frequently cited author in the included dataset, with 53 publications and 1848 citations. This research group has made sustained contributions to dynamic facial reanimation, rehabilitation, outcome assessment, and the psychosocial consequences of facial paralysis. Its recent work evaluated the psychosocial benefits of facial fillers in patients with facial nerve paralysis, suggesting an additional therapeutic option for managing facial asymmetry.^[26]^ In contrast, Terzis JK was the most frequently co-cited author and had the highest co-citation TLS, indicating a lasting influence on the shared knowledge base of nerve transfer and dynamic facial reanimation.^[18]^ *Plastic and Reconstructive Surgery* ranked 1st in both publication output and co-citation frequency, while *Otology & Neurotology* and the *Journal of Plastic, Reconstructive & Aesthetic Surgery* were also major publication sources (Tables 5 and 6). This distribution confirms that reconstructive surgery and otologic management have constituted long-standing intellectual pillars of PFP treatment research. Half of the 10 most frequently co-cited journals were classified as *Q*1 journals. Although *The New England Journal of Medicine* had the highest impact factor among these journals (IF = 96.3), its co-citation prominence is more appropriately interpreted as reflecting the disproportionate influence of landmark clinical trials than a sustained publication focus on PFP. Collectively, these findings demonstrate that publication productivity and intellectual influence represent related but distinct dimensions of journal contribution.

### 4.2. Research trends and hotspots

By integrating keyword co-occurrence, clustering, temporal overlay, and reference co-citation analyses, this study identified the principal research hotspots and emerging trends in PFP therapy. The 4 keyword clusters broadly represented etiology and acute pharmacological management, surgical intervention, dynamic nerve and muscle reanimation, and rehabilitation and outcome assessment. Similarly, the 8 reference co-citation clusters encompassed free gracilis muscle transfer, intractable PFP, Bell palsy, treatment strategies, antiviral therapy, nonflaccid facial paralysis, psychological aspects, and low-level laser therapy. Earlier studies predominantly focused on antiviral agents, corticosteroids, and muscle transfer, whereas nerve transfer and rehabilitation approaches, including acupuncture and electrical stimulation, have gained greater visibility in recent years. Taken together, these patterns indicate an expansion rather than a simple replacement of therapeutic approaches: evidence for acute pharmacological treatment has progressively consolidated, reconstructive strategies for persistent paralysis have become more refined, and increasing attention has been directed toward sequela management and long-term functional restoration.

#### 4.2.1. Acute pharmacological management

Firstly, pharmacological treatment of acute Bell palsy constituted an early knowledge foundation of PFP therapy, particularly research on corticosteroids and antiviral agents. “Antiviral therapy” and “prednisolone” were positioned toward earlier periods in the keyword overlay visualization, although prednisolone remained one of the most frequent treatment-related keywords. References with the strongest citation bursts included the landmark randomized trials conducted by Sullivan et al and Engström et al,^[22,23]^ together with the Cochrane review by Salinas et al.^[24]^ Collectively, these studies established the benefit of early corticosteroid treatment for Bell palsy while indicating that the additional benefit of antiviral therapy was limited or remained uncertain.^[27,28]^ The hypotheses of viral reactivation and intraneural edema in the pathogenesis of PFP provided the biological rationale for investigating antiviral agents and corticosteroids.^[29,30]^ The subsequent incorporation of major randomized controlled trial findings into clinical guidelines further consolidated their co-citation influence and established these studies as a durable evidence base for acute management.^[8,9]^

The declining novelty of corticosteroid- and antiviral-related terms in recent years should not be interpreted as a reduction in their clinical importance. Instead, it more likely reflects the maturation of this research question after pivotal trials and clinical guidelines had clarified their role in routine practice.^[31,32]^ The relatively recent average appearance of certain corticosteroid-related terms in the overlay map may reflect continuing guideline updates, systematic reviews, and subgroup analyses rather than the recent introduction of corticosteroid therapy itself. More importantly, most highly co-cited evidence concerning acute pharmacological treatment was derived from patients with idiopathic Bell palsy, whereas the present bibliometric dataset encompassed PFP of heterogeneous etiologies. Therefore, although PFP therapy has developed into a broad research field, its most influential evidence for acute treatment remains concentrated in a relatively specific etiological subgroup. This distinction limits the direct extrapolation of Bell palsy evidence to PFP arising from other causes and highlights the need for etiologically stratified research.

#### 4.2.2. Reconstructive surgery and dynamic facial reanimation

Reconstructive surgery and dynamic facial reanimation represented another enduring research hotspot. “Reanimation” and “surgery” were among the most frequent keywords, while free gracilis muscle transfer constituted the largest reference co-citation cluster. This finding was consistent with the central positions occupied by reconstructive surgical journals and influential surgical researchers in the author and journal networks. The sustained prominence of this theme reflects an important clinical reality: acute pharmacological therapy cannot restore dynamic facial movement in patients with severe facial nerve injury, prolonged denervation, or loss of functional facial musculature.^[33,34]^ In patients with pronounced oral commissure asymmetry, free functional gracilis muscle transfer has been reported to improve oral commissure position and facial expression symmetry and remains one of the most widely used techniques for dynamic muscle reanimation in contemporary practice.^[35–37]^ Alongside free gracilis transfer, lengthening temporalis myoplasty has emerged as another dynamic reanimation procedure that can achieve voluntary smile restoration in a substantial proportion of patients and may alleviate oral-phase swallowing dysfunction associated with facial paralysis.^[38,39]^

From a temporal perspective, muscle transfer appeared relatively early, whereas nerve transfer gained greater visibility in later publications. This transition may reflect a progressive refinement of reconstructive goals rather than the replacement of 1 technique by another. Earlier studies primarily emphasized restoration of movement amplitude and overall facial symmetry, whereas more recent research has increasingly focused on axonal regeneration, reinnervation, spontaneous smiling, and objective assessment of movement quality.^[40,41]^ Available evidence indicates that hypoglossal-facial anastomosis can improve facial appearance and expression by restoring reinnervation of the facial musculature and has become one of the most widely used nerve transfer procedures for severe facial paralysis that does not recover with conservative treatment.^[42–44]^ Nevertheless, the temporal prominence of nerve transfer should not be interpreted as evidence of superiority over muscle transfer. The suitability of each reconstructive approach depends on the etiology and duration of paralysis, the viability of the native facial musculature, donor nerve availability, and the patient’s functional priorities. The bibliometric findings therefore suggest that reconstructive research is increasingly aligning treatment selection with individual neuromuscular status and patient-defined functional goals.

#### 4.2.3. Rehabilitation and management of persistent dysfunction

By overlaying the keyword clustering and keyword time-zone maps, rehabilitation therapy was found to have emerged as an increasingly prominent research hotspot, particularly electrical stimulation and acupuncture (Fig. 6). This growing interest has coincided with greater attention to persistent functional impairments, including synkinesis, facial asymmetry, impaired muscular coordination, and reduced quality of life, which cannot be fully addressed by acute pharmacological treatment (Fig. 7C). Approximately 30% of patients continue to experience synkinesis, impaired muscular coordination, or facial asymmetry despite acute treatment^.[25]^ These residual problems cannot be adequately managed using corticosteroids or antiviral agents alone, and some patients may not be suitable candidates for reconstructive surgery. Rehabilitation may improve facial neuromuscular control and functional recovery and is increasingly incorporated into PFP management because of its generally low risk and repeatability.^[45–47]^ Developments in biofeedback, surface electromyography, and rehabilitation assessment technologies have also improved the feasibility and objectivity of related research,^[48,49]^ while the absence of a standardized treatment strategy for chronic PFP sequelae has created an important area for further investigation.

Nevertheless, the recent appearance of a keyword reflects research activity rather than clinical effectiveness or certainty of evidence. Electrical stimulation and acupuncture are frequently investigated rehabilitation modalities in PFP treatment,^[50,51]^ but small sample sizes, heterogeneous treatment parameters, inconsistent comparators, and variable outcome measures have prevented the establishment of standardized protocols and consistent endorsement in international clinical guidelines.^[52,53]^ Their bibliometric prominence should therefore not be equated with proven therapeutic efficacy. Instead, it identifies an area of substantial clinical interest in which methodological heterogeneity and variable trial quality continue to limit translation into routine practice.

#### 4.2.4. Multidimensional outcome assessment and patient-centered care

The increasing prominence of long-term functional and patient-centered outcomes represents a further change in the research architecture of PFP therapy. “Synkinesis” and “rehabilitation” were among the most frequent keywords, while nonflaccid facial paralysis and psychological aspects formed independent co-citation clusters (Table 7 and Fig. 7B). In addition, the H-B facial nerve grading system was the most frequently co-cited reference, while the Sunnybrook Facial Grading System and Facial Clinimetric Evaluation Scale were also represented within the highly cited knowledge base. These findings indicate that outcome measurement is not merely an ancillary methodological issue but a central component of knowledge development in PFP therapy.

The H-B scale is practical for grading overall facial nerve function, but its broad categories have limited sensitivity to regional motor impairment and subtle functional changes and cannot precisely quantify synkinesis or dynamic asymmetry. As a clinician-rated motor scale, it also does not fully capture patient-perceived changes in appearance or psychosocial burden.^[4,54]^ The emergence of an independent psychological cluster therefore suggests that outcome assessment is expanding from clinician-rated motor function toward a multidimensional framework incorporating patient experience and quality of life. At the same time, the use of different outcome measures across surgical and rehabilitation studies reduces comparability between interventions. These findings suggest that developing new therapeutic approaches alone is insufficient; such innovation must be accompanied by standardized and responsive measures of facial function and patient-perceived benefit.

### 4.3. Future perspectives

Our bibliometric findings indicate that research on PFP therapy has progressively expanded from acute disease control toward long-term functional restoration; however, several important research gaps remain. Addressing these gaps is essential for improving therapeutic effectiveness, patient-centered outcomes, and the clinical applicability of emerging interventions. First, future pharmacological research should adopt etiologically and stage-stratified treatment strategies. The most influential evidence for acute treatment remains concentrated in idiopathic Bell palsy,^[8,9,22–24]^ whereas PFP encompasses heterogeneous etiologies and clinical phenotypes. Future studies should therefore identify the etiologic, severity, and prognostic subgroups most likely to benefit from anti-inflammatory or antiviral therapy and clarify the optimal treatment window, dosage, and combination regimen. Second, personalized facial reanimation and prognostic assessment should be prioritized. Prospective multicenter comparative studies are needed to define the stage-specific indications and optimal timing of nerve transfer, free functional muscle transfer, and regional muscle transfer.^[55–57]^ Treatment decisions should integrate disease duration, etiology, severity, native facial muscle viability, donor nerve availability, synkinesis, and patient-defined functional goals. In parallel, prognostic models incorporating electrodiagnostic findings, imaging features, and quantitative facial-motion measurements should be developed and externally validated to improve patient selection and treatment timing. Future studies should also evaluate long-term facial movement, complications, spontaneous smiling, and psychosocial benefits. Third, increasingly investigated rehabilitation approaches, including acupuncture, electrical stimulation, biofeedback, and neuromuscular retraining, require more rigorous clinical evaluation. Adequately powered, prospectively registered multicenter trials should standardize treatment timing, intervention intensity, stimulation parameters, practitioner training, comparator selection, adherence assessment, adverse-event reporting, and follow-up duration. Fourth, a core outcome set should be developed alongside stronger international and interdisciplinary collaboration. This outcome set should integrate clinician-rated facial function, objective dynamic symmetry, synkinesis, patient-reported facial disability, quality of life, psychosocial outcomes, adverse events, and outcomes assessed at standardized long-term follow-up points, thereby improving comparability across pharmacological, surgical, and rehabilitation studies.^[58–60]^ International registries, shared data platforms, and interdisciplinary networks involving otolaryngology, neurology, plastic and reconstructive surgery, rehabilitation medicine, and psychology should also be established to reduce regional fragmentation and facilitate external validation. Addressing these priorities may transform the current intervention-centered and methodologically heterogeneous literature into a stage-oriented, personalized, and more broadly generalizable evidence framework for PFP management.

### 4.4. Strengths and limitations

The principal strength of this study is that, to our knowledge, it is the 1st comprehensive bibliometric analysis of the overall research landscape and knowledge structure of PFP therapy. By combining publication trends with analyses of collaboration networks, keyword evolution, reference co-citation, and citation bursts, we examined the field from several complementary perspectives. These analyses revealed a broad shift from an earlier emphasis on acute disease control toward increasing attention to long-term functional recovery. They also placed acute management, reconstructive surgery, rehabilitation, and patient-centered outcomes within a common analytical framework, helping to clarify the relationships among these research domains and to identify priorities for future investigation.

Several limitations should nevertheless be acknowledged. First, the data were obtained exclusively from the WOSCC and were restricted to English-language articles and reviews. Relevant publications indexed only in other international or regional databases, non-English literature, local journals, conference proceedings, and other document types may therefore have been omitted, potentially underestimating contributions from non-English-speaking countries and locally oriented research communities. Second, the broad search strategy adopted to maximize retrieval sensitivity may have included some publications only indirectly related to treatment, while studies indexed exclusively under highly specific intervention terms may have been missed. Third, restricting the analysis to publications from 2004 to 2024 may have underrepresented earlier intellectual foundations and the most recent developments. Moreover, because the search was completed on November 3, 2024, publication output for 2024 was incomplete and should not be interpreted as indicating a decline in research activity. Citation-based indicators are also influenced by publication age, journal visibility, self-citation, and cumulative network effects, while recent publications have had less time to accumulate citations. In addition, this study did not perform study-level risk-of-bias assessment or formal grading of evidence, and the inclusion of heterogeneous PFP etiologies, disease stages, and severity levels within a common network may have obscured subtype-specific patterns. Accordingly, the present bibliometric maps should be interpreted as representations of scientific attention and knowledge organization rather than as rankings of therapeutic efficacy or direct clinical recommendations.

## 5. Conclusion

In summary, this bibliometric analysis is the 1st to provide a comprehensive overview of research related to the treatment of PFP. The relevant literature published over the past 2 decades was analyzed, revealing an increasing trend in publications related to the treatment of PFP. The author and journal that have contributed the most to this field of research are Hadlock TA and *Plastic and Reconstructive Surgery*, with the latter also being the most commonly co-cited journal; the most commonly co-cited author is Terzis JK. This study systematically examined prevailing research hotspots and identified prospective directions for future investigation within the field. PFP therapy research has evolved from an early emphasis on acute pharmacological control, particularly corticosteroids and antiviral agents for Bell palsy, toward a broader continuum encompassing facial reanimation, rehabilitation, synkinesis management, and patient-centered long-term outcomes. The United States and several specialized institutions remain central to the field, whereas uneven international connectivity and heterogeneous outcome measures constrain evidence comparability. The recent visibility of nerve transfer, acupuncture, electrical stimulation, and rehabilitation reflects growing scientific interest rather than established comparative efficacy. Future research should therefore stratify patients by etiology and disease stage, undertake prospective multicenter comparisons, develop and adopt a core outcome set, and strengthen international and interdisciplinary data sharing. In summary, our study provides scholars with insights into the history and current trends of PFP treatment, offering a certain reference for further exploration in this field.

## Acknowledgments

This work was supported by the Major Research and Development Program of the Anhui Provincial Science and Technology Department (grant no. 2022E07020026); Anhui Clinical Medical Research Transformation Project (grant No. 202304295107020123) and the Clinical Research Project of Anhui University of Chinese Medicine (grant No. 2024LC083).

## Author contributions

**Conceptualization:** Fan Dai.

**Data curation:** Fangyuan Xu, Xiaoyu Han.

**Funding acquisition:** Hongliang Cheng.

**Methodology:** Fan Dai, Xiaoyu Han.

**Project administration:** Peijia Hu, Hongliang Cheng.

**Resources:** Fan Dai.

**Supervision:** Hongliang Cheng.

**Validation:** Fangyuan Xu, Peijia Hu.

**Visualization:** Xiaoyu Han.

**Writing – original draft:** Fan Dai, Fangyuan Xu.

**Writing – review & editing:** Peijia Hu, Hongliang Cheng.
